# Inositol hexaphosphate promotes intestinal adaptation in short bowel syndrome via an HDAC3-mediated epigenetic pathway

**DOI:** 10.29219/fnr.v67.8694

**Published:** 2023-02-02

**Authors:** Weipeng Wang, Ying Wang, Ying Lu, Xinbei Tian, Shanshan Chen, Bo Wu, Jun Du, Yongtao Xiao, Wei Cai

**Affiliations:** 1 Department of Pediatric Surgery, Xin Hua Hospital, School of Medicine, Shanghai Jiao Tong University, Shanghai, China; 2 Division of Pediatric Gastroenterology and Nutrition, Xinhua Hospital, School of Medicine, Shanghai Jiao Tong University, Shanghai, China; 3 Shanghai Institu of Pediatric Research, Xin Hua Hospital, School of Medicine, Shanghai Jiao Tong University, Shanghai, China; 4 Shanghai Key Laboratory of Pediatric Gastroenterology and Nutrition, Shanghai, China

**Keywords:** inositol hexaphosphate, short bowel syndrome, intestinal adaptation, HDAC3, cell proliferation

## Abstract

**Background:**

Short bowel syndrome (SBS) has high morbidity and mortality rates, and promoting intestinal adaptation of the residual intestine is a critical treatment. Dietary inositol hexaphosphate (IP6) plays an important role in maintaining intestinal homeostasis, but its effect on SBS remains unclear. This study aimed at investigating the effect of IP6 on SBS and clarified its underlying mechanism.

**Methods:**

Forty male Sprague–Dawley rats (3-week-old) were randomly assigned into four groups (Sham, Sham + IP6, SBS, and SBS + IP6 groups). Rats were fed standard pelleted rat chow and underwent resection of 75% of the small intestine after 1 week of acclimation. They received 1 mL IP6 treatment (2 mg/g) or sterile water daily for 13 days by gavage. Intestinal length, levels of inositol 1,4,5-trisphosphate (IP3), histone deacetylase 3 (HDAC3) activity, and proliferation of intestinal epithelial cell-6 (IEC-6) were detected.

**Results:**

IP6 treatment increased the length of the residual intestine in rats with SBS. Furthermore, IP6 treatment caused an increase in body weight, intestinal mucosal weight, and IEC proliferation, and a decrease in intestinal permeability. IP6 treatment led to higher levels of IP3 in feces and serum, and higher HDAC3 activity of the intestine. Interestingly, HDAC3 activity was positively correlated with the levels of IP3 in feces (*r* = 0.49, *P* = 0.01) and serum (*r* = 0.44, *P* = 0.03). Consistently, IP3 treatment promoted the proliferation of IEC-6 cells by increasing HDAC3 activity *in vitro*. IP3 regulated the Forkhead box O3 (FOXO3)/Cyclin D1 (CCND1) signaling pathway.

**Conclusion:**

IP6 treatment promotes intestinal adaptation in rats with SBS. IP6 is metabolized to IP3 to increase HDAC3 activity to regulate the FOXO3/CCND1 signaling pathway and may represent a potential therapeutic approach for patients with SBS.

## Popular scientific summary

- Inositol hexaphosphate (IP6) treatment improved intestinal adaptation of the residual intestine in rats with short bowel syndrome.
- IP6 treatment increased the levels of inositol 1,4,5-trisphosphate (IP3), activated histone deacetylase 3 (HDAC3), and regulated the Forkhead box O3 (FOXO3)/Cyclin D1 (CCND1) signaling pathway.
- IP3 promoted the proliferation of the intestinal epithelial cells by activating HDAC3 and regulated the FOXO3/CCND1 signaling pathway *in vitro.*

Short bowel syndrome (SBS), usually caused by a massive small bowel resection (SBR), is characterized by the inability of the residual intestine to adequately absorb sufficient water and nutrients to meet caloric, fluid, and electrolyte demands, thus, necessitating dependence on parenteral nutrition (PN) (1, 2). Although long-term PN is currently essential for survival and growth in neonates with SBS and intestinal failure, it can cause prolonged hospitalization and miscellaneous complications (1). Therefore, SBS is associated with high morbidity and mortality rates (3, 4). Following SBR, the residual intestine of patients with SBS undergoes a physiological process known as intestinal adaptation, causing bowel lengthening, both in length and diameter, and villous hyperplasia (5–7). The promotion of intestinal adaptation is essential for restoring enteral autonomy and reducing long-term dependence on PN support. Thus, the primary goal of treatment of patients with SBS is to promote intestinal adaptation by using the gastrointestinal tract as much as possible (8, 9). Developing ideal treatments to promote intestinal adaptation is important for improving the survival rate and quality of life of patients with SBS.

Inositol hexaphosphate (IP6, also known as phytate) is a natural fiber-associated dietary component enriched in nuts, beans, and grains (10). IP6 is rapidly metabolized into secondary phosphorylated forms in the intestine. Previous studies have demonstrated that IP6 and its secondary phosphorylated forms play crucial roles in cellular functions, including cell growth, endocytosis, migration, differentiation, and apoptosis (11–14). Oral administration of IP6 analogs attenuates intestinal inflammation and promotes survival in mouse models of Clostridioides difficile infection (15). IP6 treatment can alleviate intestinal mucosal injury and inhibit colon inflammation (16, 17). A recent study demonstrated that ingestion of dietary IP6 can alleviate intestinal inflammation caused by dextran sodium sulphate (DSS) and promote the growth of colonoids (18). These studies suggest that dietary IP6 may play an important role in maintaining intestinal homeostasis. However, to date, there is no study investigating the effect of IP6 on SBS. Therefore, the purpose of this study was to evaluate the effect of IP6 administration on intestinal adaptation in SBS and determine its underlying mechanisms.

## Materials and methods

### Animals

Three-week-old male Sprague–Dawley rats were purchased from the Shanghai Jihui Laboratory Animal Care Co. Ltd. (Shanghai, China). Rats were housed individually in an animal care facility under temperature- and humidity-controlled conditions with a 12-h light-dark cycle. Rats were provided with free access to water and fed standard pelleted rat food (Lab Mice Diet, Cat#1010085, Jiangsu Xietong Pharmaceutical Bio-engineering, Jiangsu, China). The dietary composition contained energy of 3,656 kcal/kg, protein of 22.8%, fat of 13.8%, and carbohydrate of 63.4%. The 1 kg diet contained nutrients as follows: protein, 209 g; fat, 49 g; crude fiber, 21 g; crude ash, 54 g; calcium, 12 g; phosphorus, 7.7 g; and methionine and cystine, 10.8 g. All animal experiments were approved by the Animal Research Committee of Xinhua Hospital affiliated with Shanghai Jiao Tong University School of Medicine (XHEC-F-2020-008).

### SBS model and experimental design

Forty rats were divided randomly into the following four groups (*n* = 10 per group): Sham group (underwent bowel transection), Sham + IP6 group (underwent bowel transection and treated with IP6), SBS group (75% SBR), and SBS + IP6 group (75% SBR and treated with IP6). The operative procedure and postoperative care were performed after 1 week of acclimation as described previously (19). For SBS rats, approximately 75% of the small intestine was carefully removed, including the small bowel, from 10 cm distal to the ligament of Treitz to 15 cm proximal to the ileocecal valve. Rats in the Sham group underwent bowel transection and re-anastomosis without resection at 15 cm proximal to the ileocecal junction. Following the operation, rats were fasted for 24 h and fed a regular diet and water ad libitum.

From day 1 to 13 following the operation, all rats received daily oral gavage. Rats in the Sham + IP6 and SBS + IP6 groups were administered with IP6 (Cat# P8810-10G, Merck, USA) at a dose of 2 mg/g daily. The drug dosage for rats was selected according to the effectivity of the drug dose administered in mice in the previous literature (18) and further converted to the dose in rats according to the guidelines of the Food and Drug Administration (FDA) calculator (20). The Sham and SBS groups were administered with sterile water as a control. Body weights were monitored daily.

### Sample collection

Rats were sacrificed on day 14 following the operation after overnight fasting. The portal plasma, total small intestine, and colon were rapidly harvested. Two centimeters of each intestinal sample were fixed in a 4% neutral-buffered formalin for further histological examination. The mucosa was obtained by scraping the internal intestinal mucosa after opening the intestines along the longitudinal axis.

### Intestinal morphologic assay

The residual small intestine from the ligament of Treitz to the cecum was removed. Immediately following removal of the mesentery, the residual small intestine was measured with no tension applied, and its total length proximal and distal to the anastomosis were measured. Additionally, the length of the colon was measured.

### Histological analysis

The intestinal tissues were fixed with 4% paraformaldehyde, embedded in paraffin wax, sliced, and stained with hematoxylin and eosin (HE). The villus height, crypt depth, and muscular layer thickness of 10 vertically well-oriented villi and crypts were determined using an analysis system (NIS-elements suite; Nikon) by two experienced observers blinded to the treatment.

### Intestinal epithelial cell proliferation

Crypt cell proliferation was detected by immunohistochemistry (IHC) using Ki67 as a marker of active cell division as previously described (21). The intestinal sections were dehydrated using xylol and ethanol. After blocking with 3% bovine serum albumin (BSA), sections were incubated with the Ki67 antibody (Cat# GB11141, Servicebio, China) at a dilution of 1:500 overnight in a moist chamber. The tissues were washed with Tris-buffered saline and incubated with a secondary antibody. Nuclei were counterstained with 4,6′-diamidino-2-phenylindole. The sections were further visualized using the Leica application suite and a LeicaDFC310 FX Digital Color Camera (Leica). The crypt cell proliferation rate was counted as the number of Ki67-positive cells present among 10 consecutive well-oriented crypts per slide.

### Intestinal permeability

Intestinal permeability was assessed using fluorescein isothiocyanate-labeled dextran with a molecular weight of 4,000 Da (FD4, Cat# 46944, Merck, USA) following published methods (22). Following an overnight fast, each rat was administered 1 mL of FD4 solution (5 mg/mL) by oral gavage. Following 2 h of FD4 administration, the portal vein blood was collected and centrifuged (1,660 × g, 15 min) to obtain serum. Furthermore, the FD4 concentrations of serum were measured with a fluorescence spectrophotometer (excitation at 490 nm and emission at 520 nm).

### Glucagon-like peptide-2 (GLP-2) enzyme-linked immunosorbent assay

The concentration of GLP-2 in the serum was measured using an enzyme-linked immunosorbent assay (ELISA) kit (Cat# CSB-E14203r, Cusabio, China) following the manufacturer’s instructions.

### Inositol 1,4,5-trisphosphate (IP3) ELISA assay

The fecal pellet was homogenized in cold phosphate-buffered saline (PBS), and the extract was collected following centrifugation at 4°C. IP3 ELISA was performed on the fecal extract and serum according to the manufacturer’s instructions (Cat# CSB-E13004r, Cusabio, China).

### Cell culture and treatment

The small intestinal epithelial cell line (IEC-6) cells of rat were cultured in Dulbecco’s Modified Eagle’s Medium (DMEM, Cat# G4510, Servicebio, China) supplemented with 1% streptomycin/penicillin solution (Cat# G4003, Servicebio, China) and 10% fetal bovine serum (Cat# 10091148, Thermo Fisher Scientific, USA). Cells were maintained in a humidified incubator having 95% air and 5% carbon dioxide at 37°C. Cells were serum-deprived for 4 h, and all experiments were performed in a serum-free medium. IP3 (Cat# 850115P, Merck, USA) at varying concentrations (0–10 μM) and butyrate (Cat# M41001-1, Epigentek, USA) at a final concentration of 0–10 mM were added to the medium to investigate its impact on their growth of IEC-6 cells.

### Cell proliferation assay

Cell proliferation was measured using the Cell Counting Kit-8 (CCK-8) assay (Cat# 40203ES60, YEASEN, China), according to the manufacturer’s protocol. The IEC-6 cells were seeded at equal density in 96-welled plates and incubated with IP3 or butyrate for the indicated time points. Following the treatment, the culture medium was removed and replaced with 100 μL of fresh medium containing 10 μL of CCK-8 solution in each well, and the cells were incubated at 37°C for 2 h. The number of viable cells was determined by reading the absorbance at 450 nm using a Synergy H1 Multi-Mode Reader and compared to a background blank control.

### Histone deacetylase 3 activity assay

The level of histone deacetylase 3 (HDAC3) activity in the mucosa of the distal intestine and IEC-6 cells was quantified using an enzyme-based fluorometric HDAC3 Activity Assay Kit (Cat# K343-100, Biovision, USA), according to the manufacturer’s instructions.

### EdU incorporation assay

Cell proliferation *in vitro* was also assessed using the Cell-Light EdU DNA Cell Proliferation Kit (C103102, RiboBio, China), following the manufacturer’s instructions. Following treatment, IEC-6 cells were incubated with DMEM supplemented with 50 μM EdU for 2 h at 37°C. The cells were fixed with 4% paraformaldehyde and incubated with glycine. After permeabilization with 0.5% Triton X-100, the cells were incubated with 1× Apollo^®^ staining solution for 30 min and washed with 0.5% Triton X-100 thrice, followed by a 10 min incubation in Hoechst solution. Images were captured using a laser scanning confocal microscope. The EdU-positive rates (%) were calculated as EdU-positive cell numbers/Hoechst-stained cell numbers ×100.

### Oxygen consumption rate assay

The oxygen consumption rate (OCR) of IEC-6 cells was measured using the XF Cell Mito Stress Test Kit (Cat# 103015-100, Agilent, USA), as described previously (23, 24). Analysis was performed in an XF assay medium under basal conditions and followed by the addition of PBS or IP3 (1 μM), oligomycin (1 μM), carbonyl cyanide 4-(trifluoromethoxy) phenylhydrazone (FCCP, 0.75 μM), rotenone, and antimycin A (0.5 μM).

### Western blotting

Nuclear and cytoplasmic protein extraction was performed using a nuclear and cytoplasmic protein extraction kit (Cat# MP1551-100T, MKBio, China), according to the manufacturer’s instructions. Protein concentrations were determined using a bicinchoninic acid protein assay kit (Thermo Fisher Scientific, USA). Equal amounts of nuclear protein for each sample (30 μg) were separated by sodium dodecyl sulphate-polyacrylamide gel electrophoresis and transferred to nitrocellulose membranes. Immunodetection was performed using an enhanced chemiluminescence ECL system (Cat# 36222ES60, Yeason, China). Histone H3 served as the loading control. The primary antibodies used in this study included anti-histone H3 antibody (1:1000, Cat# ab1791, Abcam, UK), anti-cyclin D1 (1:200, Cat# ab16663, Abcam, UK), HDAC3 antibody (1:1000, Cat# AF5349-50 μL, Affinity, China), anti-histone H3 (acetyl K9) antibody (1:500, Cat# ab32129, Abcam, UK), and FoxO3a (75D8) rabbit mAb (1:1000, Cat# 2497S, Cell Signaling Technology, USA). The secondary antibodies were goat anti-rabbit IgG HRP-linked antibody (1:2000, Cat# 7074S, Cell Signaling Technology, USA) and anti-mouse IgG HRP-linked antibody (1:2000, Cat# 7076S, Cell Signaling Technology, USA). The relative optical density of the protein bands was measured using the ImageJ software, and the protein levels were normalized to histone H3.

### Quantitative real-time polymerase chain reaction

Total RNA was extracted from the mucosa samples using the RNeasy Mini kit (Cat#74101, Qiagen, Germany), according to the manufacturer’s instructions. The levels of the genes were detected using the High-Capacity cDNA Reverse Transcription kit (Cat# 4368813, Thermo Fisher Scientific, USA) and SYBR-Green Universal Master Mix kit (Cat# A25742, Thermo Fisher Scientific, USA). A list of real-time polymerase chain reaction (PCR) primers is provided in Supplementary Table 1.

### Statistical analysis

Results are indicated as mean ± standard error of the mean (SEM). All analyses were performed using the GraphPad Prism software version 8.0.1 (GraphPad, San Diego, CA). The differences between the two groups were analyzed using the unpaired two-tailed Student’s *t*-test or Mann–Whitney U test. One-way analysis of variance (ANOVA) with post-hoc Bonferroni’s multiple-comparison analysis or Kruskal–Wallis rank-sum test with Dunn’s multiple-comparison test was performed to compare the differences among multiple groups. The correlation between HDAC3 activity and levels of IP3 was assessed by linear Pearson correlation analysis. *P* < 0.05 was considered significant.

## Results

### IP6 improves intestinal adaptation in the SBS rat model

**Fig. 1:**
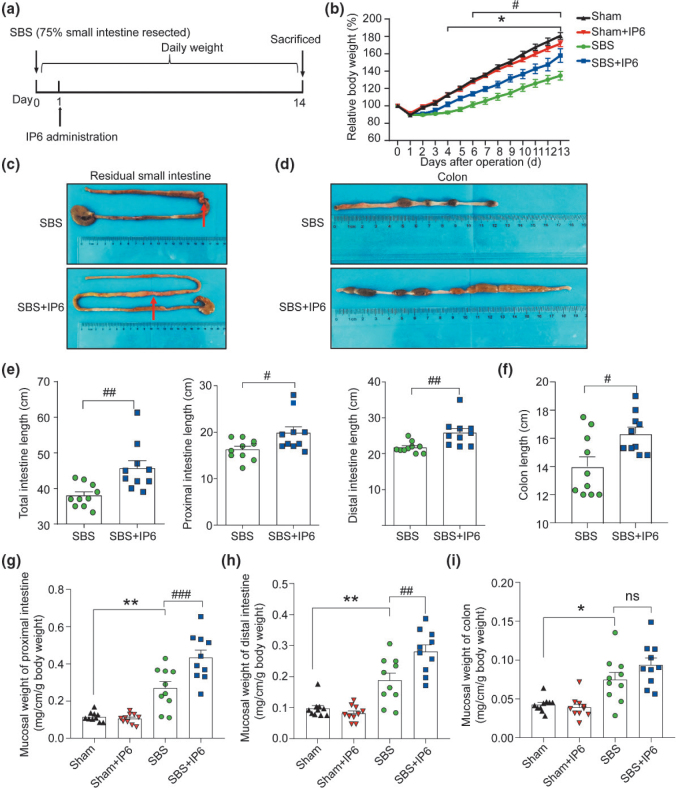
IP6 increases the intestinal length and mucosal weight in the SBS rats. (a) Schematic illustration of the experimental approach of the SBS rats’ model. (b) Comparing body weight change of rats in the different groups. (c) Macroscopic view of the residual small intestine in the SBS and SBS + IP6 group. Arrows indicate anastomosis sites. (d) Macroscopic view of the colon in the SBS and SBS + IP6 group. (e) Comparison of the length of the total, proximal, and distal small intestine between the SBS and SBS + IP6 groups. (f) Comparison of the length of colon between the SBS and SBS + IP6 groups. (g) Comparison of the mucosal weight of proximal small bowel. (h) Comparison of the mucosal weight of distal small bowel. (i) Comparison of the mucosal weight of the colon. Values are mean ± SEM, *n* = 10 per group; ns, not significant; * *P* < 0.05, ** *P* < 0.01, SBS group versus Sham group; ^#^ *P* < 0.05, ^##^ *P* < 0.01, ^###^ *P* < 0.001, SBS + IP6 group versus SBS group.

To investigate the effects of IP6 treatment on SBS rats, rats were treated with IP6 by oral gavage daily starting from day 1 following operation (Fig. 1a). A significant weight loss was observed from day 4 following operation in the SBS group compared with the Sham group (Fig. 1b). And rats in the SBS + IP6 group exhibited a higher body weight compared to those in the SBS group (Fig. 1b). Representative images of the residual small intestine and colon are presented in Fig. 1c and d. Compared with those in the SBS group, the total residual small intestine in the SBS + IP6 group was longer (118%, Fig. 1c and e). Both the proximal small intestine and distal small intestine in the SBS + IP6 group were longer than those in the SBS group (Fig. 1c and e). Additionally, the colon was longer in the SBS + IP6 group than those in the SBS group (117%, Fig. 1d and f).

Compared to rats in the Sham group, rats in the SBS group had a greater mucosal weight of the proximal small intestine (Fig. 1g), distal small intestine (Fig. 1h), and colon (Fig. 1i). IP6 treatment in SBS rats caused an additional increase in the weight of the intestinal mucosa. Compared to the rats in the SBS group, those in the SBS + IP6 group presented an additional increase in the mucosal weight of the proximal intestine (Fig. 1g) and distal intestine (Fig. 1h).

**Fig. 2:**
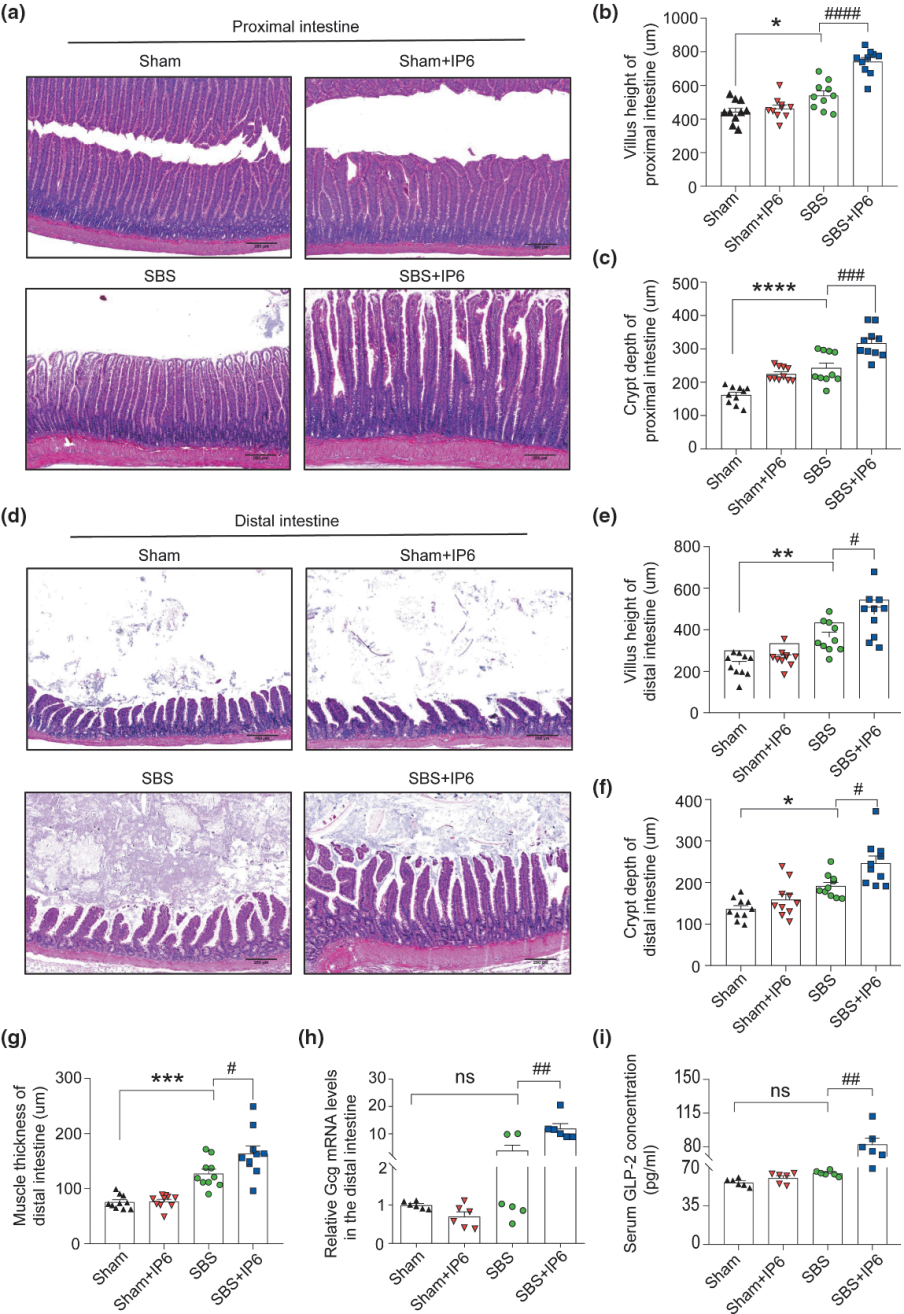
IP6 improves histological adaptation of the residual intestine in the SBS rats. (a) Representative images of hematoxylin-eosin (HE) staining of the proximal small intestine of rats in different groups (*n* = 10 per group). Scale bars, 250 μm. (b) Comparison of villus height in the proximal small intestine of rats in different groups (*n* = 10 per group). (c) Comparison of crypt depth in the proximal small intestine of rats in different groups (*n* = 10 per group). (d) Representative images of HE staining of the distal small intestine of rats in different groups (*n* = 10 per group). Scale bars, 250 μm. (e) Comparison of villus height in the distal small intestine of rats in different groups (*n* = 10 per group). (f) Comparison of crypt depth in the distal small intestine of rats in different groups (*n* = 10 per group). (g) Comparison of muscle thickness in the distal small intestine of rats in different groups (*n* = 10 per group). (h) The relative mRNA expression levels of *Gcg* of the mucosa in the distal small intestine (*n* = 6 per group). Values were normalized to *Actb* expression. (i) The levels of glucagon-like peptide-2 (GLP-2) in the serum were evaluated by ELISA analysis (*n* = 6 per group). Values are mean ± SEM; ns, not significant; * *P* < 0.05, ** *P* < 0.01, *** *P* < 0.001, **** *P* < 0.0001, SBS group versus Sham group; ^#^ *P* < 0.05, ^##^ *P* < 0.01, ^###^ *P* < 0.001, ^####^ *P* < 0.0001, SBS + IP6 group versus SBS group.

As revealed by histological staining, in the proximal intestine, a significant increase was observed in villus height (1.37-fold, Fig. 2a and b) and crypt depth (1.31-fold, Fig. 2a and c) in the SBS + IP6 group compared with the SBS group. Similarly, in the distal intestine, IP6 treatment caused an additional intestinal growth in SBS rats (Fig. 2d), which was characterized by increased villus height (1.30-fold, Fig. 2e), crypt depth (1.28-fold, Fig. 2f), and muscle thickness (1.29-fold, Fig. 2g). Compared with the SBS group, the mRNA expression levels of glucagon (*Gcg*) in the residual intestinal mucosa and concentrations of GLP-2 in the serum were increased in the SBS + IP6 group (Fig. 2h and i).

### IP6 promotes the proliferation of IECs

**Fig. 3:**
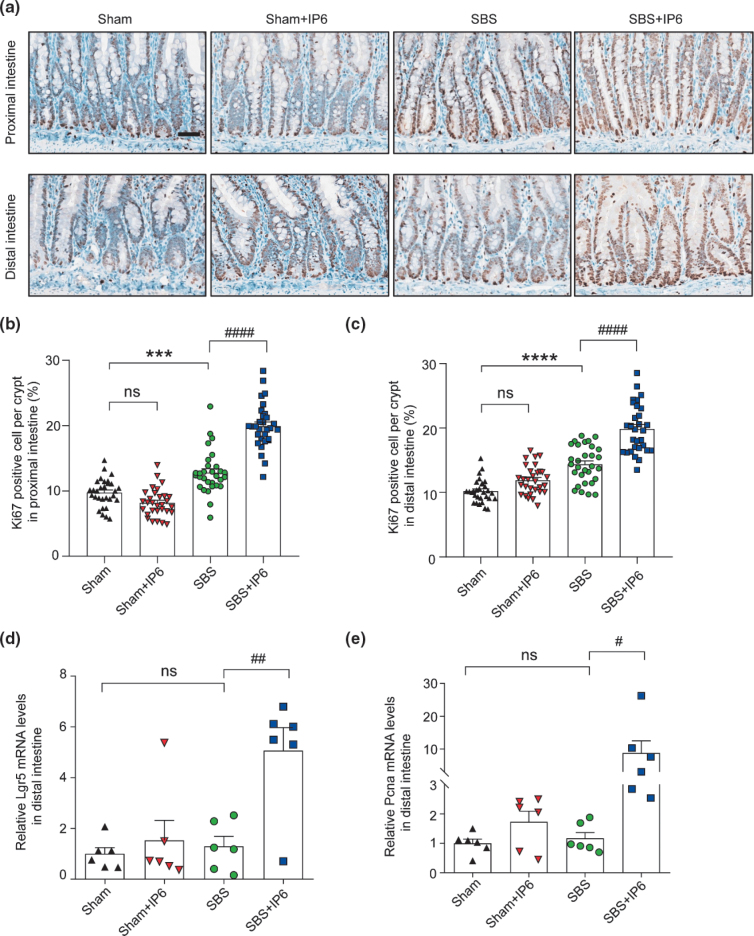
IP6 promotes intestinal proliferation in the SBS rats. (a) Representative images of immunohistochemistry (IHC) staining for Ki67 in the residual small intestine (upper panel showing the IHC of the proximal small intestine; lower panel showing the IHC of the distal small intestine). Scale bars, 50 μm. (b) The number of Ki67 positive cells per crypt of the proximal small intestine was quantified (*n* = 3 per group, 10 crypts were analyzed by one rat). (c) The number of Ki67 positive cells per crypt of the distal small intestine was quantified (*n* = 3 per group, 10 crypts were analyzed by one rat). (d) The relative mRNA expression levels of *Lgr5* in the distal small intestine (*n* = 6 per group). Values were normalized to *Actb* expression. (e) The relative mRNA expression levels of *Pcna* in the distal small intestine (*n* = 6 per group). Values were normalized to *Actb* expression. Values are mean ± SEM; ns, not significant; *** *P* < 0.001, **** *P* < 0.0001, SBS group versus Sham group; ^#^ *P* < 0.05, ^##^ *P* < 0.01, ^####^ *P* < 0.0001, SBS + IP6 group versus SBS group.

In the proximal and distal small intestine, Ki67-positive cells were rarely detected in the Sham group, a few positive cells were detected in the SBS group, and several positive cells were detected in the SBS + IP6 group (Fig. 3a). Quantitatively, in the proximal small intestine, the proportion of Ki67-positive cells per crypt was higher in the SBS + IP6 group than those in the SBS group (1.56-fold, Fig. 3b). In the distal small intestine, the proportion of Ki67-positive cells per crypt was also higher in the SBS + IP6 group than those in the SBS group (1.38-fold, Fig. 3c). Consistently, the mRNA expression levels of leucine rich repeat containing G protein-coupled receptor 5 (*Lgr5*) and proliferating cell nuclear antigen (*Pcna*) were markedly increased in the SBS + IP6 group than those in the SBS group (Fig. 3d and e).

### IP6 decreases intestinal permeability

**Fig. 4:**
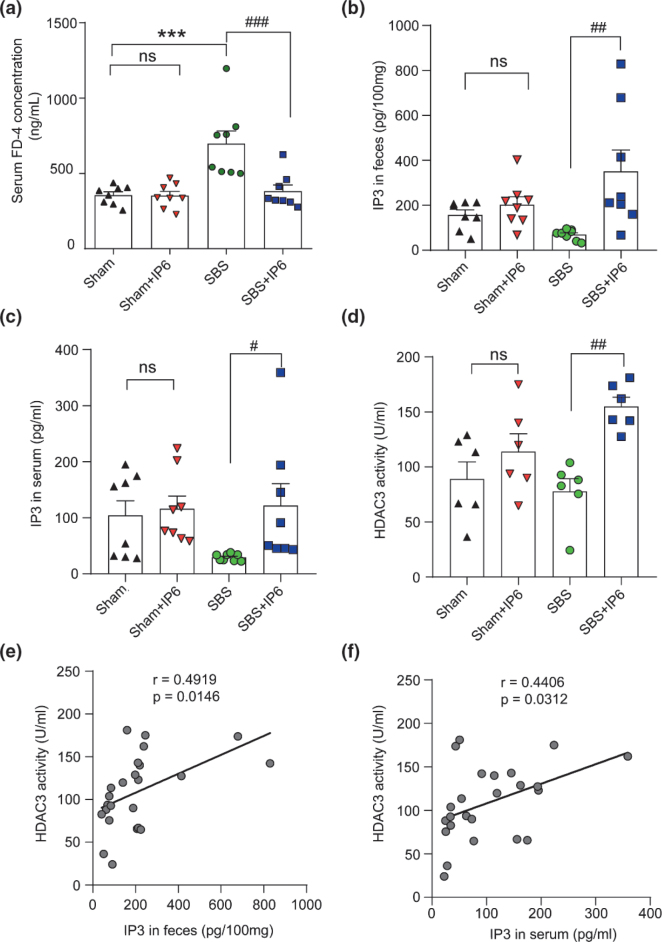
IP6 increases IP3 levels and HDAC3 activity in the intestine of rats. (a) Serum concentration of FD4 of rats in different groups (*n* = 8 per group). (b) The levels of IP3 in the feces were determined by ELISA analysis (*n* = 8 per group). (c) The levels of IP3 levels in the serum were determined by ELISA analysis (*n* = 8 per group). (d) HDAC3 activity of the distal intestine was detected by HDAC3 activity assay (*n* = 6 per group). (e) Correlation analysis of HDAC3 activity with the levels of IP3 in the feces. (f) Correlation analysis of HDAC3 activity with the levels of IP3 in the serum. Values are mean ± SEM; *** *P* < 0.001, SBS group versus Sham group; ^#^ *P* < 0.05, ^##^ *P* < 0.01, ^###^ *P* < 0.001, SBS + IP6 group versus SBS group.

There was a significant increase in intestinal permeability in the SBS rats since a higher concentration of FD4 in their serum was detected compared with the Sham group (Fig. 4a). However, IP6 treatment reduced the serum concentration of FD4 of the rats in the SBS + IP6 group by 45% compared with the rats in the SBS group, indicating that intestinal permeability was decreased following IP6 treatment (Fig. 4a).

### IP6 is metabolized to IP3 to increase HDAC3 activity in the intestine

Dietary IP6 can be metabolized to IP3 in the intestine (25). To investigate whether IP6 functions through IP3 in SBS rats, we examined the level of IP3 in the intestine among the different groups. Consequently, compared with those in the SBS group, the levels of IP3 in feces and serum were significantly increased in the SBS + IP6 group (Fig. 4b and c), supporting that IP6 was metabolized into IP3 in the intestine.

To test whether IP6 treatment affects HDAC3 activity, we evaluated HDAC3 activity in the intestinal mucosa of rats among the different groups. The results indicated that rats in the SBS + IP6 group had a significantly higher activity of HDAC3 compared with those in the SBS group (Fig. 4d). Importantly, correlation analysis demonstrated that HDAC3 activity was positively correlated with the levels of IP3 in feces (*r* = 0.49, *P* = 0.01, Fig. 4e) and serum (*r* = 0.44, *P* = 0.03, Fig. 4f).

### IP3 promotes the proliferation of IECs by increasing HDAC3 activity in vitro

**Fig. 5:**
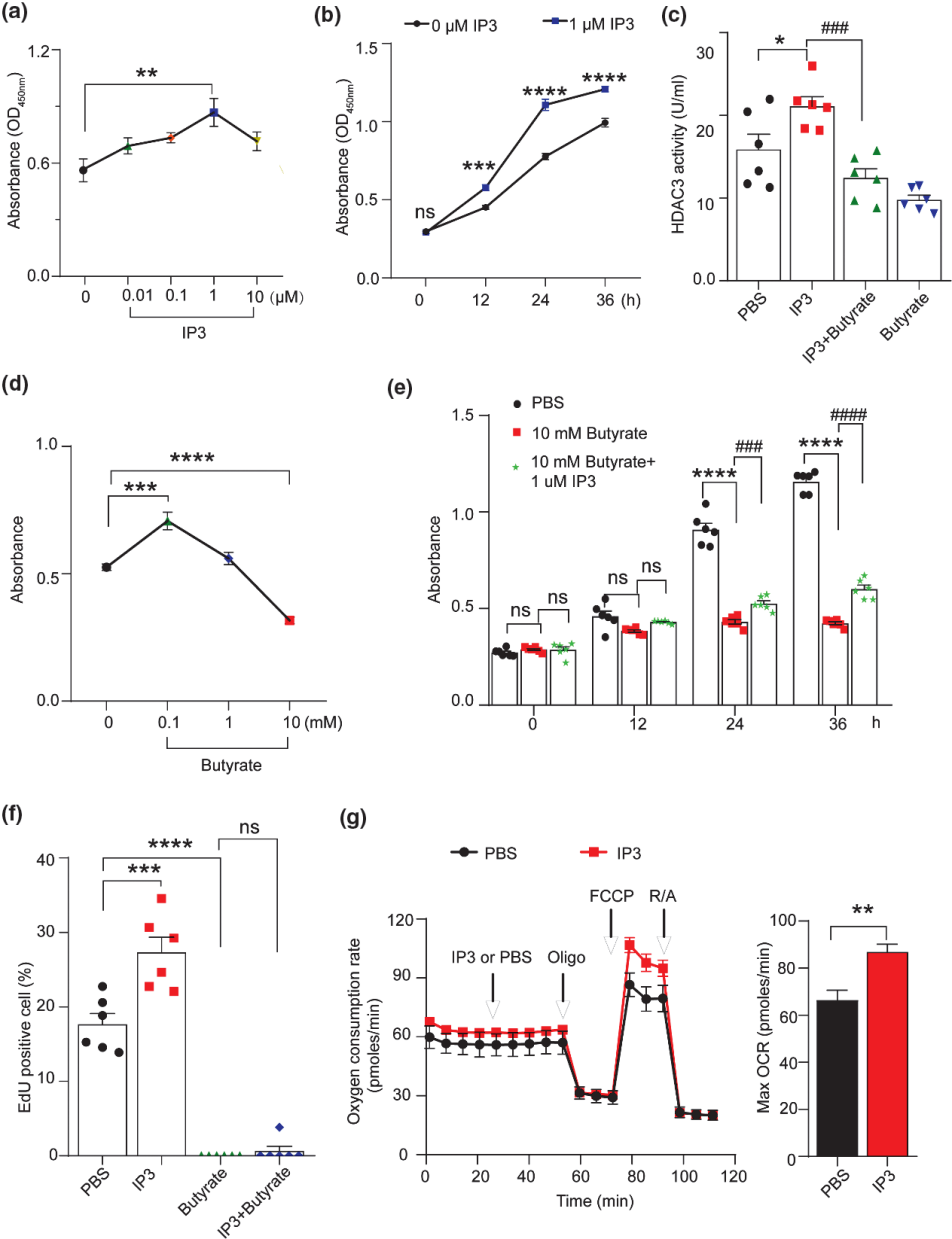
IP3 stimulates cell proliferation by increasing HDAC3 activity in IEC-6 cells. (a) Cell proliferation of IEC-6 treated with IP3 at different concentrations for 24 h was detected using the CCK-8 assay (*n* = 6 per group). ** *P* < 0.01, 1 μM IP3 group versus 0 μM IP3 group. (b) Cell proliferation of IEC-6 cells treated with 1 μM IP3 at indicated time points was detected using the CCK-8 assay (*n* = 6 per group). *** *P* < 0.001, **** *P* < 0.0001, 1 μM IP3 group versus 0 μM IP3 group. (c) HDAC3 activity of IEC-6 cells was detected using HDAC3 activity assay in different groups. IEC-6 cells were cocultured with PBS, 1 μM IP3, 10 mM butyrate+1 μM IP3, and 10 mM butyrate (*n* = 6 per group). * *P* < 0.05, IP3 group versus PBS group; ^###^ *P* < 0.001, IP3 group versus IP3+Butyrate group. (d) Cell proliferation of IEC-6 cells treated with butyrate at different concentrations for 24 h (*n* = 6 per group). *** *P* < 0.001, **** *P* < 0.0001, butyrate group versus PBS group. (e) Cell proliferation of IEC-6 cells treated with PBS, 10 mM butyrate, and 10 mM butyrate+1 μM IP3 at indicated time points was detected using the CCK-8 assay (*n* = 6 per group). **** *P* < 0.0001, butyrate group versus PBS group; ^###^ *P* < 0.001, ^####^ *P* < 0.0001, butyrate group versus IP3+butyrate group. (f) DNA synthesis of IEC-6 cells was measured by the EdU incorporation assay at 24 h after treatment with PBS, 1μM IP3, 10mM butyrate, and 10mM butyrate + 1μM IP3. The fraction of EdU-positive IEC-6 cells was counted and plotted. *** *P* < 0.001, **** *P* < 0.0001, versus PBS group. (g) Effect of IP3 on the OCR of IEC-6 cells. The OCR was measured at basal conditions and after PBS or IP3, oligomycin (Oligo), carbonyl cyanide-4-(trifluoromethoxy), phenylhydrazone (FCCP), and rotenone/antimycin (R/A) injections (*n* = 5 – 6). ** *P* < 0.01 versus PBS group. Values are mean ± SEM; ns, not significant.

We further investigated the role of IP3 and HDAC3 activity in the proliferation of IEC-6 cells. First, to examine whether IP3 affects the growth of IEC-6 cells, we incubated the cells with different concentrations of IP3 for 24 h. The proliferation of IEC-6 cells was accelerated following IP3 treatment in a dose-dependent manner, and a significant growth-promoting effect was observed when the cells were treated with IP3 at a concentration of 1 μM (Fig. 5a). Additionally, the CCK-8 assay revealed that the absorbance signal increased immediately after 24 h in the IP3-treated group compared with the unstimulated (IEC-6 cells alone) time-matched controls (Fig. 5b).

Second, we detected HDAC3 activity in IEC-6 cells. The results revealed that HDAC3 activity was increased in the IP3-treated group compared with the control group (Fig. 5c), suggesting that IP3 could increase HDAC3 activity in IEC-6 cells. Third, to test the functional importance of HDAC3 activity for IP3-mediated cell proliferation, we treated IEC-6 cells with 10 mM butyrate (an HDAC3 inhibitor). When IEC-6 cells were treated with 10 mM butyrate, HDAC3 activity was inhibited (Fig. 5c). Additionally, HDAC3 activity increased by IP3 was attenuated by butyrate treatment (Fig. 5c). Last, to test whether butyrate treatment affects the proliferation of IEC-6 cells, we incubated the cells with different concentrations of butyrate for 24 h. Consequently, cell proliferation was significantly inhibited by 10 mM butyrate (Fig. 5d). However, the growth-suppressing effect of 10 mM butyrate can be rescued by IP3 treatment (Fig. 5e). Quiescent IEC-6 cells incubated with 1 μM IP3 for 24 h demonstrated a distinct increase in the incorporation of EdU compared with the control group (Fig. 5f and Supplementary Fig. 1). However, incubation with 10 mM butyrate caused a significant reduction in EdU incorporation in IEC-6 cells (Fig. 5f and Supplementary Fig. 2). Moreover, we monitored the OCR of IEC-6 cells and observed a significant increase in OCR upon IP3 treatment (Fig. 5g).

### IP6 promotes intestinal adaptation by regulating the HDAC3/FOXO3/CCND1 signaling pathway

**Fig. 6:**
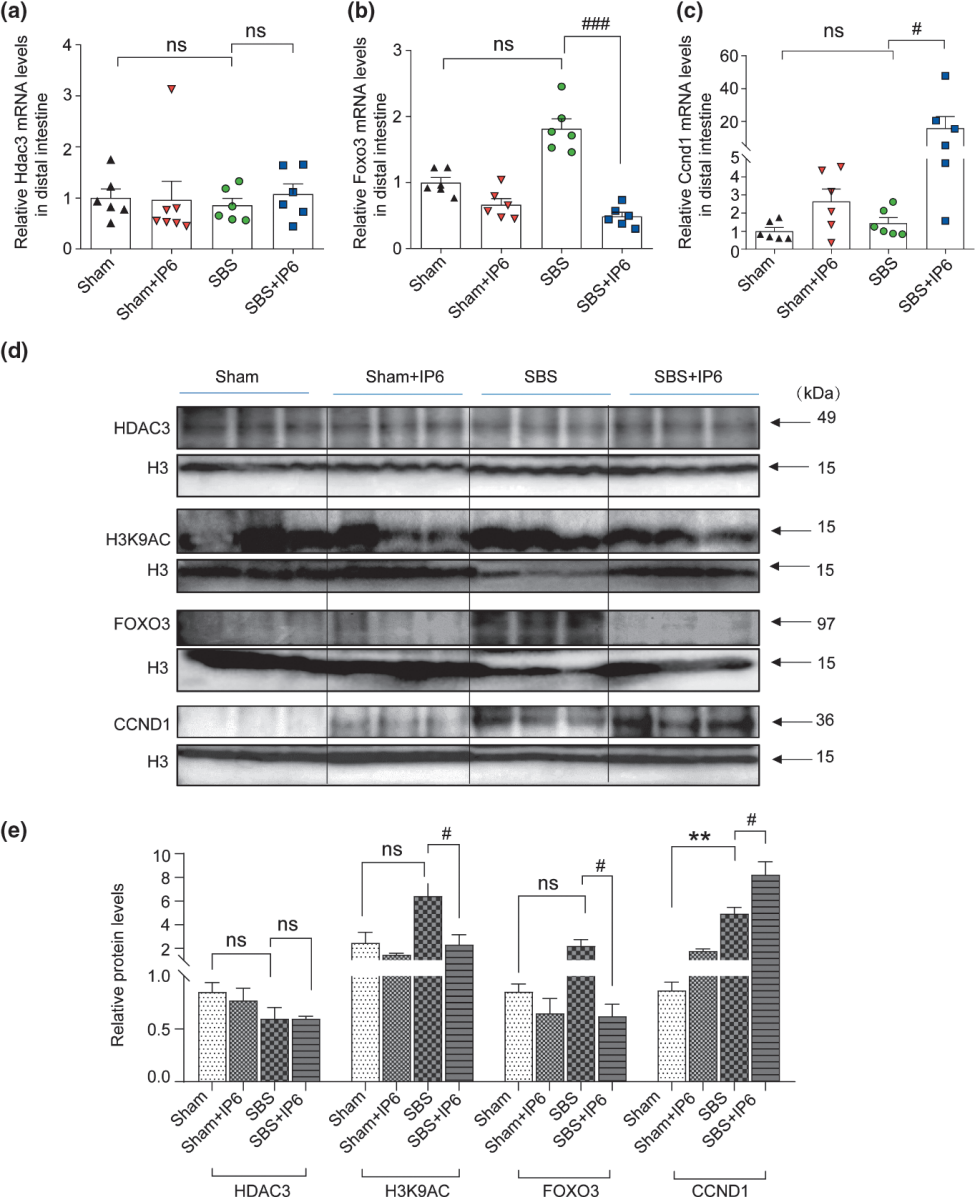
IP6 regulates the FOXO3/CCND1 signaling pathway in rats. (a) The relative mRNA expression levels of *Hdac3* in the distal small intestine (*n* = 6 per group). Values were normalized to *Actb* expression. (b) The relative mRNA expression levels of *Foxo3* in the distal small intestine (*n* = 6 per group). Values were normalized to *Actb* expression. (c) The relative mRNA expression levels of *Ccnd1* in the distal small intestine (*n* = 6 per group). Values were normalized to *Actb* expression. (d) Western blotting analysis of the mucosa of the distal small intestine was performed to detect the protein expression levels of HDAC3, H3K9AC, FOXO3, and CCND1. Histone H3 was used as an internal control. (e) Relative protein expression of HDAC3, H3K9AC, FOXO3, and CCND1 of the mucosa in the distal small intestine. Values are mean ± SEM; ns, not significant; ** *P* < 0.01, SBS group versus Sham group; ^#^ *P* < 0.05, ^###^ *P* < 0.001, SBS + IP6 group versus SBS group.

We measured the mRNA expression levels of six genes that have been reported to be important for cell proliferation, including *Hdac3*, *Forkhead box O3* (*Foxo3*), *Cyclin D1* (*Ccnd1*), *Epidermal growth factor* (*Egf*), *Insulin-like growth factor* (*Igf*), and *Yes-associated protein* (*Yap1*) using quantitative real-time polymerase chain reaction (qPCR) in the intestine of rats (Fig. 6a–c and Supplementary Fig. 2a–c). Consequently, the mRNA expression levels of *Hdac3*, *Egf*, and *Igf* were not affected by IP6 treatment, albeit the mRNA expression levels of *Foxo3* significantly decreased and *Ccnd1* significantly increased in the SBS + IP6 group compared with the SBS group (Fig. 6a–c and Supplementary Fig. 3a–c). Western blot analysis revealed that the protein expression levels of HDAC3 were not affected by IP6 treatment (Fig. 6d and e). Consistent with the increase in HDAC3 activity induced by IP6 treatment (Fig. 4d), the protein expression levels of H3K9AC, which is the established target for HDAC3 at directly repressed genes, were downregulated in the SBS + IP6 group than those in the SBS group (Fig. 6d and e). The protein expression levels of FOXO3 were downregulated, and for CCND1, they were upregulated in the SBS + IP6 group than those in the SBS group (Fig. 6d and e).

**Fig. 7:**
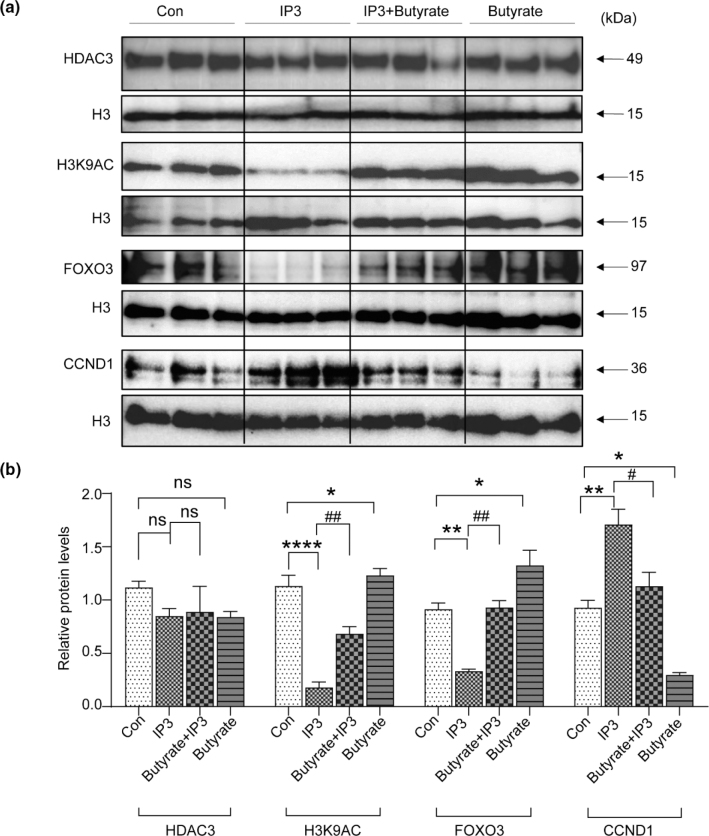
IP3 regulates the FOXO3/CCND1 signaling pathway in IEC-6 cells. (a) Western blotting analysis of IEC-6 cells was performed to detect the protein expression levels of HDAC3, H3K9AC, FOXO3, and CCND1. Histone H3 was used as an internal control. (b) Relative protein expression of HDAC3, H3K9AC, FOXO3, and CCND1 of the mucosa in the distal small intestine. Values are mean ± SEM; ns, not significant; * *P* < 0.05, ** *P* < 0.01, **** *P* < 0.0001, SBS group versus Sham group; ^#^ *P* < 0.05, ^##^ *P* < 0.01, SBS + IP6 group versus SBS group.

Furthermore, we explored whether IP6 metabolism regulated the FOXO3/CCND1 pathway by altering HDAC3 activity. Therefore, we tested HDAC3 activity and the protein expression levels of HDAC3 in the presence of IP3 or butyrate *in vitro*. IP3 and butyrate treatment altered HDAC3 activity albeit did not alter the protein levels of HDAC3 (Fig. 5c and 7a, b). The protein expression levels of H3K9AC were downregulated by IP3 albeit upregulated by butyrate (Fig. 7a and b). Consistent with the *in vivo* findings, protein expression levels of FOXO3 were downregulated, and CCND1 was upregulated by IP3 treatment (Fig. 7a and b). In contrast, butyrate treatment upregulated protein levels of FOXO3 and downregulated protein levels of CCND1 (Fig. 7a and b).

## Discussion

Previous studies have demonstrated that dietary IP6 plays an important role in maintaining intestinal homeostasis; however, its role in the intestinal adaptation of SBS is still unknown. In this study, we demonstrated that IP6 treatment promoted adaptation of the residual intestine in SBS. IP6 treatment increased the length of the residual intestine, improved the intestinal mucosal barrier, and increased the proliferation of IECs. IP6 is metabolized into IP3 in the intestine, which increases HDAC3 activity and promotes the proliferation of IECs. Additionally, we observed that the FOXO3/CCND1 signaling pathway was regulated by IP3.

Intestinal adaptation following SBR is a compensatory response involving morphological and functional changes, which increase the absorptive surface area and improve absorption (6, 7). These changes include increased villus height and crypt depth, increased bowel caliber, increased number of IECs, intestinal epithelial hyperplasia, and increased expression of epithelial transporter proteins (7). Consistent with previous studies, this study demonstrated that SBR caused an increased villus height, crypt depth, and muscular thickness. Correspondingly, the SBS rats presented a higher mucosal weight in the residual intestine than the Sham control group. Intriguingly, further increases in the villus height, crypt depth, and muscular thickness were observed in the IP6-treated SBS rats compared with the SBS rats. Additionally, the mucosal weight of the residual small intestine was increased by IP6 treatment. More importantly, IP6 treatment increased the length of the residual small intestine and colon. These results strongly suggested that the absorptive surface area of the residual intestine was increased by IP6 treatment in the SBS rats. Intestinal morphology is an indicator of intestinal health and absorption capability (26). Nutritional absorption and digestion are both improved by better intestinal morphology. Weight gain is a result of improved adaptive intestinal functionalities in SBS rats. In this study, the body weight of SBS rats was increased by IP6 treatment. These findings suggested that intestinal absorption may be improved by IP6 treatment. It is well-known that increased hormone secretion is a change in intestinal adaptation. GLP-2 is an intestinal hormone released by enteroendocrine L-cells of the distal ileum and colon (27). In piglets and humans with intestinal resection, GLP-2 treatment has been reported to increase intestinal adaptation, reduce fecal output, and allow weaning of PN (27–29). In this study, an increase was observed in the expression levels of *Gcg* and serum concentrations of GLP-2 in the IP6-treated SBS rats. It suggests that IP6 treatment promotes the secretion of GLP-2 in the residual intestine. As previously reported, Ki67 staining of the residual intestine revealed that SBR could cause a higher IEC proliferative activity (21). IP6 treatment further increased the proportion of Ki67-positive cells per crypt in the residual intestine. Consistently, the mRNA expression levels of *Lgr5* and *Pcna* increased by IP6 treatment in the SBS rats. These indicate that IEC proliferation of the residual intestine was enhanced by IP6 treatment. In line with a previous study, we observed that SBS rats had higher intestinal permeability (22). Previous studies have indicated that IP6 has favorable effects on the intestinal mucosal barrier (16, 17). Similarly, in this study, IP6 treatment reduced the intestinal permeability induced by SBR. Collectively, these results demonstrated that IP6 treatment promoted intestinal adaptation in the SBS rats.

Furthermore, we investigated how IP6 treatment stimulates intestinal adaptation following SBR. IP6 can be metabolized rapidly into secondary phosphorylated forms (IP3 and other inorganic phosphates) in the intestine with the help of the enzymes phytases produced by commensal bacteria, such as *E. coli* (25). IP3 is a second messenger with important physiological functions. Structural analyses have supported that IP3 can directly bind to the inositol-phosphate-binding site in HDAC3 to increase HDAC3 activity (13, 30). HDAC3 is a critical factor that integrates commensal-bacteria-derived signals to establish normal host–commensal relationships and maintain intestinal homeostasis (31). A recent study demonstrated that IP3 can increase HDAC3 activity in the epithelium to promote tissue repair in DSS-induced colitis mice (18). Therefore, we hypothesized that IP6 is metabolized into IP3, which further increases HDAC3 activity in the intestine of SBS rats.

To test this hypothesis, we first determined whether IP6 could be metabolized into IP3. A higher concentration of IP3 in the feces and serum was observed in the IP6-treated SBS rats than those in the SBS rats, suggesting that IP6 was metabolized into IP3 in the intestine of IP6-treated SBS rats. Moreover, HDAC3 activity was increased by IP6 treatment. Correlation analysis indicated that HDAC3 activity was positively correlated with the levels of IP3. Furthermore, *in vitro*, HDAC3 activity was increased in IEC-6 cells when incubated with IP3. These data provide evidence linking IP6 metabolism and epithelial HDAC3 activity in SBS rats. To further investigate the relationship between the increased HDAC3 activity induced by IP3 and IEC proliferation, IEC-6 cells were incubated with IP3. Additionally, we selected butyrate, an HDAC inhibitor, to inhibit the activity of HDAC3. Consequently, incubation with IP3 increased HDAC3 activity and promoted the proliferation of IEC-6 cells. In contrast, butyrate inhibited HDAC3 activity and suppressed IEC-6 cell proliferation. OCR measurements presented that IP3 treatment increased mitochondrial respiration in IEC-6 cells. H3K9AC is an established target of HDAC3 (32). Consistent with the HDAC3 activity altered by IP3 and butyrate, the protein expression levels of H3K9AC were downregulated by IP3 albeit upregulated by butyrate. Interestingly, although HDAC3 activity was altered, its expression levels did not change. These results indicate that IEC proliferation enhanced by IP6 depends on the increased HDAC3 activity induced by IP3, which is metabolized from IP6 in the intestine.

Furthermore, we explored the underlying mechanism of HDAC3 activity in the regulation of IEC proliferation. Previous studies have suggested that butyrate increases the binding of FOXO3 to several key cell cycles through HDAC inhibition, thereby suppressing stem and progenitor cell proliferation in the intestine (33). Additionally, FOXO3 transcriptional activity can be regulated by HDACs (34). FOXO3 is a negative regulator of cell proliferation and can repress the activity of CCND1 (35, 36). In this study, by using qPCR and western blot analysis, we determined that IP6 treatment regulated the FOXO3/CCND1 signaling pathway, which was characterized by downregulated FOXO3 expression and upregulated CCND1 expression. Consistent with these *in vivo* results, IP3 downregulated FOXO3 and upregulated CCND1 in IEC-6 cells. In contrast, butyrate upregulated FOXO3 and downregulated CCND1 expression.

## Conclusion

This study demonstrated for the first time that IP6 treatment enhanced intestinal adaptation via an HDAC3-mediated epigenetic mechanism in rats with SBS. IP6 can be metabolized into IP3 in the intestine to increase epithelial HDAC3 activity, thereby regulating the FOXO3/CCND1 signaling pathway and promoting epithelial cell proliferation. These findings support the application of IP6 therapy to enhance adaptive intestinal growth in patients with SBS. Additionally, strategies to alter epigenetic changes may have therapeutic potential for treating SBS.

## Supplementary Material

Inositol hexaphosphate promotes intestinal adaptation in short bowel syndrome via an HDAC3-mediated epigenetic pathway

## Data Availability

The data presented in this study are available on request from the corresponding author.
