# Supplementary material for: Inositol hexaphosphate promotes intestinal adaptation in short bowel syndrome via an HDAC3-mediated epigenetic pathway
Source: Food Nutr Res. 2023 Feb 2;67:10.29219/fnr.v67.8694. doi: 10.29219/fnr.v67.8694 (PMC9899046; doi:10.29219/fnr.v67.8694)
Supplement: Inositol hexaphosphate promotes intestinal adaptation in short bowel syndrome via an HDAC3-mediated epigenetic pathway [file FNR-67-8694-s001.docx]

**Supplementary Table**

**Table S1. Primers for gene expression using real-time PCR**

| Genes |  | Sequence (5’-3’) | Size (bp) | NCBI Gene ID |
| --- | --- | --- | --- | --- |
| *Hdac3* | Forward | ATGACCAGAGTTACAAGCACCT | 74 | NM_053448.2 |
|  | Reverse | GATGCACGTCGGCTGATAGA |  |  |
| *Foxo3* | Forward | CTCCCTGCGAGTGTCTATAACTTT | 71 | NM_001106395.1 |
|  | Reverse | ATTCCTCCTCCTCCCTTCCAC |  |  |
| *Ccnd1* | Forward | TCCGGAGACCGGTCGTAGAG | 171 | NM_171992.5 |
|  | Reverse | CCGGTCGTTGAGGAGATTGG |  |  |
| *Lgr5* | Forward | GTGAGCTGGATGGCAGGATG | 184 | NM_001106784.1 |
|  | Reverse | GCATTTCCAGCAAGACGCAA |  |  |
| *Pcna* | Forward | CACGTATATGCCGGGACCTT | 88 | NM_022381.3 |
|  | Reverse | TCCCCACTCGCAGAAAACTT |  |  |
| *Gcg* | Forward | GCCATTCACAGGGCACATTC | 113 | NM_012707.2 |
|  | Reverse | GGCAATGTTGTTCCGGTTCC |  |  |
| *Egf* | Forward | GGTCCACCCATTGGCAAAAC | 118 | NM_012842.2 |
|  | Reverse | CACGAATCCTTCCCGACACA |  |  |
| *Igf1* | Forward | GGGACGTACCAAAATGAGCG | 159 | NM_001082477.2 |
|  | Reverse | ACGAACTGAAGAGCGTCCAC |  |  |
| *Yap1* | Forward | ATTTCGGCAGGCAATACGGA | 76 | XM_006242490.4 |
|  | Reverse | GCTGCGGAGAGCTAATTCCT |  |  |
| Actb | Forward | CCCGCGAGTACAACCTTCTT | 83 | NM_031144.3 |
|  | Reverse | CGCAGCGATATCGTCATCCA |  |  |

**Figure S1.** EdU incorporation assay of IEC-6 cells. DNA synthesis of IEC-6 cells was measured by the EdU incorporation assay at 24 h after treatment with PBS, 1 μM IP3, 10 mM butyrate, and 10 mM butyrate+1 μM IP3. Cell nuclei were stained with Hoechst solution (blue).


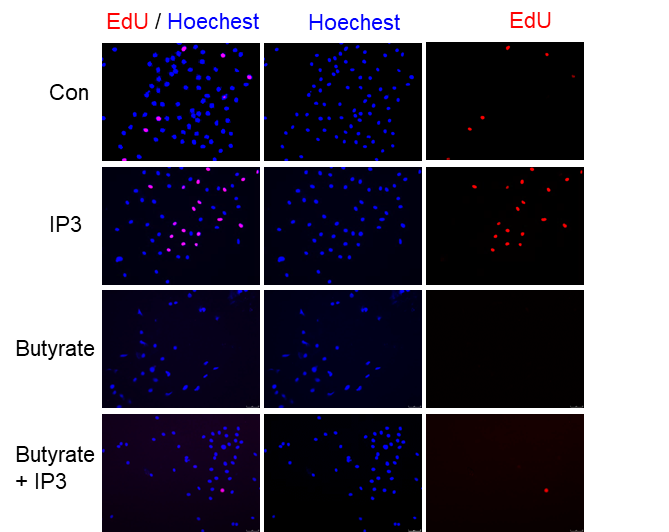


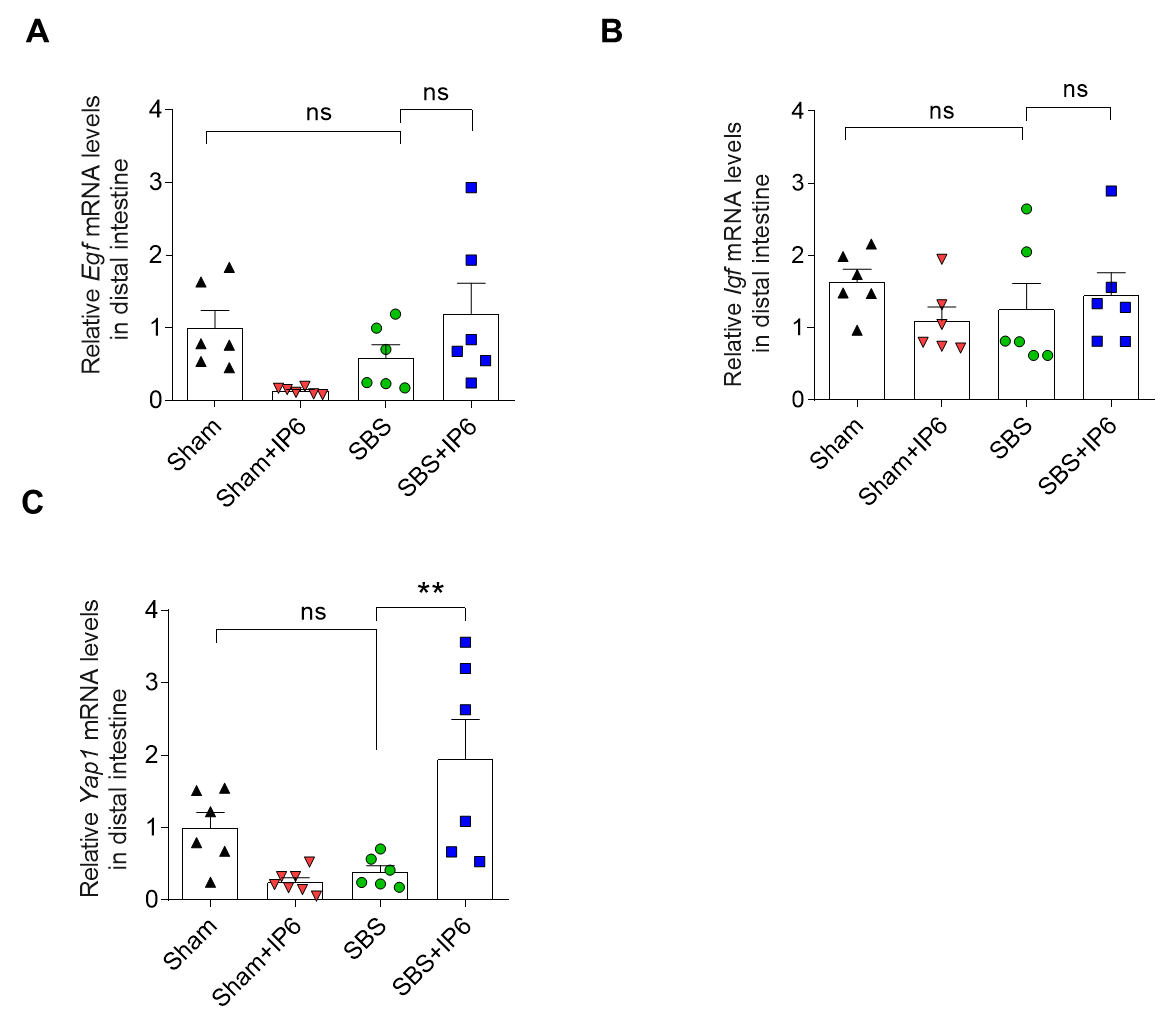
**Figure S2.** Gene expression altered by IP6. (A) The relative mRNA expression levels of *Epidermal growth factor* (*Egf*) in the distal small intestine (n = 6 per group). (B) The relative mRNA expression levels of *Insulin like growth factor* (*Igf*) in the distal small intestine (n = 6 per group). (C) The relative mRNA expression levels of *Yes associated protein* (*Yap1*) in the distal small intestine (n = 6 per group). Values were normalized to *Actb* expression. Values are mean ± SEM; one-way ANOVA with post-hoc Bonferroni’s multiple-comparison analysis was used for statistical analysis; ns, not significant; ** *p* < 0.01.
